# Immunology, Treatment and Public Health Aspects of Subarachnoid Hemorrhage

**Published:** 2020-04

**Authors:** Junjing ZHAO, Jianping ZHANG, Yongxia BU, Wei LU, Gejin ZHAO

**Affiliations:** 1. Medical Department, Binzhou Central Hospital, Bingzhou 251700, P.R. China; 2. Ward 1, Department of Neurology, Gaotang People’s Hospital, Gaotang 252800, China; 3. Central Sterile Supply Department, Binzhou People’s Hospital, Bingzhou 251700, P.R. China; 4. Cerebrovascular and Neurological Severe Cases, Linzi District People’s Hospital, Zibo 255400, P.R. China; 5. Department of Neurosurgery for Professional Clinical Medicine, Linzi District People’s Hospital, Zibo 255400, P.R. China

**Keywords:** Interventional embolization of aneurysms, Subarachnoid hemorrhage, Craniotomy, Clinical efficacy

## Abstract

**Background:**

We aimed to explore the treatment and safety of subarachnoid hemorrhage.

**Methods:**

A retrospective analysis was applied on 137 patients with subarachnoid hemorrhage treated in Binzhou Central Hospital, Bingzhou, China from March 2015 to October 2018. Seventy cases with interventional embolization of intracranial aneurysms were divided as the observation group, and 67 cases with craniotomy for aneurysm clipping were divided as the control group. The changes of immune globulins before and after surgery, CD4+, CD8+, NIHSS scores, BI scores, the total effective rate of subarachnoid hemorrhage, the total length of postoperative hospital stay and conditions of postoperative complications as well as 30-day survival were compared between the two groups.

**Results:**

The levels of Ig G, Ig M, Ig A, and CD4+ after surgery in the observation group were significantly lower than those before surgery (*P*<0.05), but significantly higher than those in the control group (*P*<0.05); the total time of postoperative hospitalization in the observation group was shorter than that in the control group (*P*<0.05). The incidence of intracranial infection and cerebral vasospasm in the observation group was significantly lower than that in the control group (*P*<0.05). The NIHSS score of the observation group was significantly lower than that of the control group (*P*<0.05), and the BI score was significantly higher than that of the control group (*P*<0.05).

**Conclusion:**

Patients with subarachnoid hemorrhage undergoing interventional embolization of aneurysms can reduce the impact on immune function, decrease the adverse reactions caused by treatments, shorten the length of hospital stay and fully improve the efficacy.

## Introduction

Subarachnoid hemorrhage (SAH) patients account for only a small fraction of total stroke patients, but nearly half of deaths in strokes are SAH patients (1, 2). SAH is estimated to have a global incidence of 6.67/100,000, and about 500,000 people worldwide suffer from subarachnoid hemorrhage every year, nearly two-thirds of the population are from low-income and middle-income countries (3–5). Nearly 10.2% of patients will be admitted to hospital after 30 days, and the 30-day mortality is as high as 40% (6–9). Usually, 35% of surviving patients have permanent disability, cognitive deficits, and some psychiatric symptoms after one-year suffering from SAH (10).

The surgical treatment methods for SAH patients are mainly craniotomy for aneurysm clipping and interventional embolization of intracranial aneurysms. Craniotomy for aneurysm clipping is more common than interventional embolization of intracranial aneurysms and the recurrence rate is also lower. Interventional embolization of intracranial aneurysms is used in treatment failure or recurrence of clipping (11–13). Aneurysm clipping has advantages and disadvantages compared with interventional embolization of aneurysms, effects of repair of clipping are better, and the clinical effect of embolization is also better (14). The immune function of patients with aneurysmal subarachnoid hemorrhage, who have undergone craniotomy and clipping, has changed significantly (15). Therefore, the recovery of postoperative immune function can be observed by detecting the level of immune globulins of patients after the surgery.

Therefore, we aimed to study the clinical efficacy and safety of interventional embolization of aneurysms and aneurysm clipping in the treatment of subarachnoid hemorrhage, to explore a better way in the treatment of subarachnoid hemorrhage and to provide clinical references.

## Materials and Methods

### Clinical Materials of Patients

Overall, 137 patients with subarachnoid hemorrhage treated in Binzhou Central Hospital from March 2015 to October 2018 were selected. Among them, 70 cases of patients treated with interventional embolization of intracranial aneurysms were regarded as the observation group, and 67 cases of patients treated with craniotomy for aneurysm clipping were regarded as the control group. In the observation group, there were 47 male patients and 23 female patients, and the mean age was (48.8±13.7) years old. There were 44 males and 23 females in the control group, with a mean age of (51.3±14.2) years.

The study was approved by the Medical Ethics Committee and all patients were informed and signed an informed consent form.

### The Inclusion and Exclusion Criteria

The inclusion criteria were as follows: According to imaging and pathology, SAH was diagnosed as the first onset; no relevant surgical treatments were performed before the study; clinical data were complete and can be followed up by telephones.

The exclusion criteria were as follows: Severe liver and renal insufficiency, patients with other malignancies, patients with other severe cardiovascular and cerebrovascular diseases, severely inflammatory patients, pregnant or nursing women, and patients with immune system defects.

### Main Instruments and Reagents

Ig G protein, Ig M protein, Ig A protein ELISA kits (Shanghai Abcam Company, China, ab100547, ab137982, ab196263).

### Methods of Treatment

Interventional embolization of aneurysms was performed on patients in the observation group. Angiography was performed under general anesthesia, and the size and position of aneurysms were confirmed, then interventional embolization was conducted with guglielmi detachable coiling (16). Craniotomy for aneurysm clipping was applied in patients of the control group. Angiography was performed under general anesthesia, and the size and position of the aneurysms were confirmed. selected the appropriate position according to the location of the aneurysms, found an aneurysm, temporarily blocked the proximal end of the aneurysm, and then aneurysm clipping was used to occlude the aneurysm (17). The same routine care was used in the two groups after surgery.

### Methods of Detection

Five ml of fasting venous blood one day before surgery, one day after surgery and five days after surgery of patients were respectively collected into blood-collection tube in the two groups, and centrifugation with 3000 rp/min was performed for 10 minutes. The serum was separated and then was collected and stored in a refrigerator at 80°C. Flow cytometry was used to detect CD4+ and CD8+. ELISA was used to detect IgG, IgM and IgA concentrations. Set blank holes, standard holes, and sample holes to be tested. Standard holes were added with 50 μL of standard samples with different concentrations, samples to be tested holes were added with 50 μL of samples to be tested, then incubated at 37 °C for 60 minutes, and washed. 100 μL of the enzyme-labeled antibody was added to each blank hole, standard hole and sample hole, the reaction hole was sealed with a sealing membrane, incubated at 37 °C for 60 minutes, and washed. 100 μL of streptavidin solution was added to each hole and incubated at 37 °C for 15 minutes, then 100 μL of TMB substrate solution was added to each hole and incubated at 37°C for 15 minutes, and 50 μL of stopping solution was added to each hole. The OD value of each hole was measured at a wavelength of 450 nm, and a standard curve was drawn to get a linear regression equation. The OD value of the sample was substituted into the equation to calculate the concentration of the sample.

### Evaluation of Efficacy

According to the patient’s postoperative digital subtraction angiography, the Raymond classification was used for evaluation of the effect. Complete occlusion (no contrast agent was observed in the aneurysm); neck residual (contrast agent was observed in the aneurysm neck); partial occlusion (Contrast agent was observed in the aneurysm cavity) (18).

### Complication assessment criteria

After surgery, the patient developed focal neurological signs or deterioration of consciousness level, and the positive results of CSF bacterial culture were intracranial infection. Doppler ultrasound was performed, the average blood flow velocity of the middle cerebral artery >120 cm / s was cerebral vasospasm. CT imaging examination was performed, and any one of the below items meant hydrocephalus. IV ventricle width > 20 mm; III ventricle width > 6 mm; bilateral posterior nucleus spacing > 25 mm; bilateral lateral ventricle frontal tip spacing >45 mm. When the volume of intracranial hematoma in CT imaging examination exceeds the total amount of residual hematoma and excretion, it is considered as rebleeding.

### Follow-up

Overall, 137 patients were followed up by telephones, visits, etc., and the follow-up time was 30 days, every five days after the 30-day postoperative period. The follow-up deadline was November 2018. The overall survival is the time from the first day after surgery to the last follow-up or death.

### Observation Indicators

Main outcome measures: Comparison of levels of Ig G, Ig M, Ig A one day before surgery, one day after surgery, and five days after surgery in the observation group and control group, postoperative Raymond grading, Glasgow Prognosis Score, total effective rates of treatment, and the 30-day survival of the patients after treatment were counted.

Secondary outcome measures: Clinical data of the two groups, total length of postoperative hospital stay, incidence of adverse reaction rate, and preoperative and postoperative NIHSS scores and BI scores.

### Statistical Analysis

This study used SPSS 20.0 (Chicago SPSS co., LTD., USA) and GraphPad Prism 7 (San Diego Graphpad Software co., LTD., USA); the chi-square test was used in the usage rate (%) of counting data, represented by x^2^; Fisher’s test was used when the number of samples was greater than or equal to 40 and the theoretical frequency was less than 1; measurement data were expressed by (Means±SEM); all measurement data were in accordance with normal distribution; independent sample t test was applied in comparison between the two groups; repeated variance analysis was applied in comparison of three or more groups, indicated by F; patients’ survival for 30 days was analyzed by K-M survival; log-rank test was analyzed and P less than 0.05 was considered as statistically significant.

## Results

### Clinical Data of Patients

There was no statistical difference between the observation group and the control group in gender, age, BMI, past medical history (hypertension, diabetes, hyperlipidemia), smoking history, alcohol history, place of residence, white blood cells, red blood cells, urea nitrogen, creatinine, clinical symptoms (headache, disturbance of consciousness, nausea and vomiting), Hunt-Hess classification (Table 1).

**Table 1:** Clinical data of patients

***Variables***	***Observation group(n=70)***	***Control group(n=67)***	***t/χ2 value***	**P *value***
Gender			0.033	0.855
Male	47(67.14)	44(65.67)		
Female	23(32.86)	23(34.33)		
Age(yr)	48.8±13.7	51.3±14.2	1.049	0.296
BMI(kg/m2)	22.15±1.82	22.04±1.97	0.340	0.735
Past medical history				
Hypertension	19(27.14)	21(31.34)	0.292	0.589
Diabetes	13(18.57)	12(17.91)	0.010	0.920
Hyperlipidemia	8(11.43)	7(10.45)	0.034	0.854
Smoking history			0.026	0.872
Yes	26(37.14)	24(35.82)		
No	44(62.86)	43(54.18)		
Alcohol history			0.119	0.731
Yes	16(22.86)	17(25.37)		
No	54(77.14)	50(74.63)		
Place of residence			0.143	0.705
City	59(84.29)	58(86.57)		
Country	11(15.71)	9(13.43)		
White blood cells (×1012/L)	12.93±3.41	13.23±3.62	0.499	0.618
Red blood cells(×1012/L)	4.67±0.93	4.45±0.88	1.421	0.158
Urea nitrogen(mmol/L)	4.17±0.72	4.21±0.76	0.316	0.752
Creatinine(μmol/L)	55.86±7.74	56.29±8.31	0.314	0.754
Clinical symptoms				
Headache	47(67.14)	42(62.69)	0.299	0.585
Disturbance of consciousness	28(32.86)	30(37.31)	0.320	0.572
Nausea and vomiting	40(57.14)	35(52.24)	0.332	0.564
Hunt-Hess classification			0.616	0.432
≤III level	48(68.57)	50(74.63)		
>III level	22(31.43)	17(25.37)		

Note: BMI: Body Mass Index

### Levels of Ig G, Ig M, Ig A before and after Treatment of Patients in the Two Groups

Levels of Ig G, Ig M, and Ig A 1 day after surgery in the observation group were significantly lower than those before surgery (*P*=0.070, *P*<0.001, *P*<0.001), and significantly higher than those in the control group (*P*=0.030, *P*<0.001, *P*<0.001). Levels of Ig G, Ig M, and Ig A 5 days after surgery in the observation group were significantly higher than those in the control group (*P*=0.026, *P*<0.001, *P*<0.001) (Tables 2–4).

**Table 2:** Ig G levels before and after treatment of patients in the two groups

***Groups***	***Ig G(g/L)***			***F***	***P***
	***Before surgery***	***One day after surgery***	***Five days after surgery***		
Observation	11.36±5.11	9.63±4.37^*^	10.87±4.86#	3.331	<0.001
group(n=70)					
Control group (n=67)	11.32±5.06	8.32±3.79^*^	9.14±4.07^*^#	13.946	<0.001
T value	0.046	2.199	2.254		
*P* value	0.963	0.030	0.026		

Note:

* indicates a difference compared with preoperative values (*P* < 0.05), and

# indicates a difference compared with values at one day after surgery (*P* < 0.05)

**Table 3:** Ig A levels before and after treatment of patients in the two groups

***Groups***	***Ig A(g/L)***			***F***	**P**
	***Before surgery***	***One day after surgery***	***Five days after surgery***		
Observation group(n=70)	2.74±0.64	2.14±0.53^*^	2.58±0.57#	31.114	<0.001
Control group(n=67)	2.68±0.61	1.53±0.32^*^	1.96±0.43^*^#	121.009	<0.001
T value	0.561	8.111	7.163		
*P* value	0.576	<0.001	<0.001		

Note:

* indicates a difference compared with preoperative values (*P* < 0.05), and

# indicates a difference compared with values at one day after surgery (*P* < 0.05)

**Table 4:** Ig M Levels Before and After Treatment of Patients in the Two Groups

***Groups***	***Ig M(g/L)***			***F***	***P***
	***Before surgery***	***One day after surgery***	***Five days after surgery***		
Observation group(n=70)	1.64±0.52	1.15±0.32^*^	1.58±0.47#	51.482	<0.001
Control group(n=67)	1.67±0.56	0.94±0.27^*^	1.23±0.39^*^#	92.115	<0.001
T value	0.046	2.199	2.254		
*P* value	0.963	0.030	0.026		

Note:

* indicates a difference compared with preoperative values (*P* < 0.05), and

# indicates a difference compared with values at one day after surgery (*P* < 0.05)

### CD4+, CD8+ levels before and after treatment in both groups

One day after surgery, the CD4+ level of the observation group was significantly lower than that of the control group (*P*<0.001), and significantly higher than that of the control group (*P*=0.146); the CD8+ level was significantly higher than that of the control group (*P*<0.001) and significantly lower than the control group (*P*=0.002). Five days after surgery, the CD4+ and CD8+ levels in the observation group were equivalent to those before surgery, and the CD4+ level was significantly higher than that in the control group (*P*=0.002), but the CD8+ level was significantly lower than that in the control group (*P*=0.043) (Table 5 and 6).

**Table 5:** CD4+ levels before and after treatment in both groups

*Groups*	***CD4+(%)***				
	***Before surgery***	***One day after surgery***	***Five days after surgery***	***F***	**P**
Observation group(n=70)	42.14±9.52	34.64±7.88^*^	39.84±8.32#	25.906	<0.001
Control group(n=67)	39.87±8.58	31.94±7.22^*^	35.27±8.14^*^#	21.765	<0.001
t value	1.464	2.088	3.248		
*P* value	0.146	0.039	0.002		

Note:

* vs Before surgery, *P* < 0.05; and

# vs one day after surgery, *P* < 0.05

**Table 6:** CD8+ levels before and after treatment in both groups

***Groups***	***CD8+(%)***				
	***Before surgery***	***One day after surgery***	***Five days after surgery***	***F***	**P**
Observation group(n=70)	31.27±8.22	35.75±7.35^*^	32.58±7.85#	12.562	<0.001
Control group(n=67)	31.95±7.78	38.22±7.02^*^	35.23±7.33^*^#	24.602	<0.001
t value	0.497	2.010	2.040		
*P* value	0.620	0.046	0.043		

Note:

* vs before surgery, *P* < 0.05; and

# vs one day after surgery, *P* < 0.05

### Comparison of postoperative efficacy evaluation between the two groups

We compared the postoperative efficacy evaluation between the two groups and found no statistical difference (Table 7).

**Table 7:** Comparison of efficacy evaluation between the two groups

	***Observation group (n=70)***	***Control group (n=67)***	***Z value***	**P *value***
Complete occlusion	54 (77.14)	46 (68.66)		
Neck residual	12 (17.14)	18 (26.87)		
Partial occlusion	4 (5.72)	3 (4.47)	1.006	0.315

### Comparison of the Total Length of Postoperative Hospital Stay of Patients Between the Two Groups

The total length of postoperative hospital stay in the observation group was found to be shorter than that of the control group, and there was a significant difference (*P*< 0.001).

### Comparison of Postoperative Complications of Patients Between the Two Groups

The incidence rate of intracranial infection and cerebral vasospasm in the observation group was significantly lower than that in the control group (*P*<0.05). There was no significant difference in the incidence rate of hydrocephalus and rebleeding between the observation group and the control group (Table 8).

**Table 8:** Comparison of postoperative complications of patients between the two groups

***Postoperative Complications***	***Observation group (n=70)***	***Control group (n=67)***	***x^2^ value***	**P *value***
Intracranial infection	0(0)	8(11.94)		0.003
Cerebral vasospasm	11(15.71)	20(29.85)	4.671	0.031
Hydrocephalus	13(18.57)	9(13.43)	0.671	0.413
Rebleeding	10(14.29)	7(10.45)	0.464	0.496

### NIHSS score and BI score before and after treatment

**Fig. 1:**
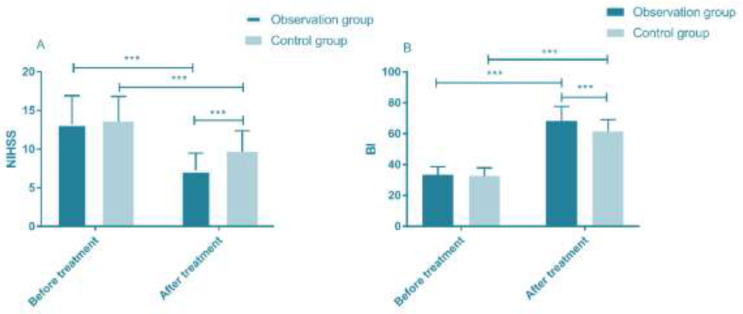
NIHSS score and BI score before and after treatment. A. There was no significant difference in NIHSS score between the two groups before treatment (t=0.487, *P*=0.627), and the observation group after treatment was significantly lower after treatment than that before treatment (t=11.945, *P* < 0.001). The NIHSS score of the control group after treatment was significantly lower than that before treatment (t=8.365, *P*<0.001), and the NIHSS score of the observation group after treatment was significantly lower than that of the control group (t=5.624, *P*<0.001). B. There was no significant difference in the BI score between the two groups before treatment (t=0.477, *P*=0.714). The BI score of the observation group after treatment was significantly lower than that before treatment (t=28.635, *P*<0.001). The BI score of the control group after treatment was significantly lower than that before treatment (t=26.958, *P*<0.001). The BI score of observation group was significantly lower than the control group after treatment (t=4.597, *P*<0.001). ^***^, *P*<0.001

The observation group NIHSS score (7.24±2.24) and the control group NIHSS score (9.64±2.74) after treatment were significantly lower than those before treatment (*P*<0.001). The observation group BI score (68.26±9.37) and the control group BI score (61.58±7.49) after treatment was significantly lower than those before treatment (*P*<0.001). The NIHSS score of the observation group after treatment was significantly lower than that of the control group (*P*<0.001), and the BI score was significantly higher than that of the control group (*P*<0.001) (Fig. 1).

### The 30-day Survival of Patients after Surgery in the Two Groups

**Fig. 2:**
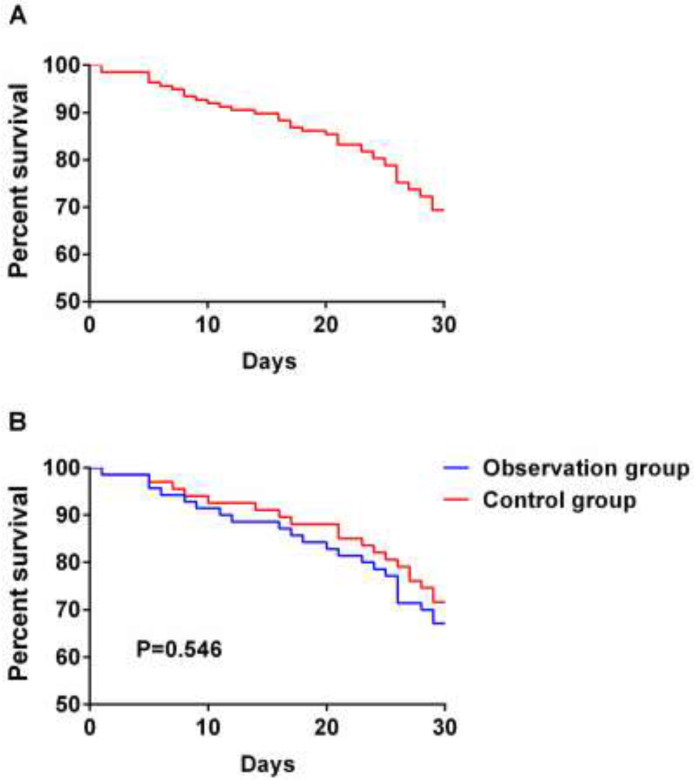
The 30-day Survival of Patients in the Two Groups A. Patients were followed up for 30 days, and the survival rate was 68.61%. B. The 30-day survival rate was 67.14% in the observation group and 71.64% in the control group. There was no statistical difference in the 30-day survival of the two groups (*P*=0.546)

In 30 days, 43 patients died and 94 survived, with a survival rate of 68.61%; in the observation group, 23 patients died and 47 patients survived, with a survival rate of 67.14%; in the control group, 19 patients died and 48 patients survived, with a survival rate of 71.64%. It was found that there was no statistical difference in 30-day survival of patients between the two groups by plotting the K-M survival curve (Fig. 2).

## Discussion

At present, some studies suggest that when patients develop SAH, peripheral immune cells are activated in damaged tissues and they enter the brain parenchyma to release inflammatory cytokines, which immediately leads to neuroinflammation and thus enlarges damage (19). Therefore, for SAH patients, if operative treatments can be performed as soon as possible after admission, they may obtain better recovery (20, 21). Interventional embolization is a mainly minimally invasive surgery, with a high risk of arterial coagulation, embolization and dissection (19). Aneurysm clipping needs a craniotomy surgery that may lead to some postoperative complications. Lai et al (22) used single factor and multivariate logistic regression analysis to investigate hypothetical risk factors to determine independent prognostic factors, and when the shape of aneurysms is irregular or more complex, it will also increase the incidence rate of complications after aneurysm clipping (23, 24). We compared the postoperative complications of patients in the two groups. The results may indicate that patients undergoing interventional embolization are less likely to develop intracranial infections and cerebral vasospasm than patients with aneurysm clipping.

In this study, we also compared the levels of Ig G, Ig M, Ig A, CD4+ and CD8+ of patients in the two groups. We believe that this result mainly involves embolization, which only requires the introduction of embolic agents from the blood vessels, but arterial embolization requires craniotomy; patients with interventional embolization have a greater probability of infection, therefore, the recovery time of immune function is longer than that of interventional embolization. Zhang et al (25) analyzed the difference in total hospital stay between unruptured aneurysms involved in curly occlusion and aneurysm clipping, and he revealed that the total hospital stay of unruptured aneurysms involved in curly occlusion was much shorter than that of aneurysm clipping, which was similar to our conclusion that patients with interventional embolization had shorter total hospital stay. After that, digital subtraction angiography was performed, and Raymond grading was used to evaluate the efficacy of the patients. The results suggest that interventional embolization has better efficacy on patients and is more suitable as the preferred treatment method for SAH patients. There was no difference in the one-year morbidity and death rate between clipping and curly embolization (26). That research was longer than ours, which further supports our results that two surgeries have no impact on patients’ survival.

At present, with the development of medical technology, there are some new embolic materials. Fiorella, et al (27) reported a new intracavitary implant such as a pinpeline embolization device (PED). Compared with the situation that traditional coil embolization often leads to incomplete occlusion or recanalization of aneurysms months after treatment, PED plays a role in shunting aneurysms and creating an environment conducive to thrombosis. Over time, the PED is incorporated into the vascular wall. At last, aneurysms are permanently excluded from the cerebrovascular systems and eventually rebuilds the diseased mother artery.

In this article, we have not compared long-term efficacy and adverse reactions. We did not discuss the correlation between other corresponding clinical indicators and subarachnoid hemorrhage. This also needs to be discussed in subsequent trials. Finally, we do not know whether there are differences in the efficacy between new embolic materials such as PED and traditional embolic materials, so we hope to study the differences between them in future experiments.

## Conclusion

Patients with subarachnoid hemorrhage undergoing interventional embolization of aneurysms can reduce the impact on immune function, decrease the adverse reactions caused by treatments, shorten the length of hospitalization stay and fully improve the efficacy, compared with those undergoing aneurysm clipping. But the impact of the two on the 30-day survival is indistinguishable.

## Ethical considerations

Ethical issues (Including plagiarism, informed consent, misconduct, data fabrication and/or falsification, double publication and/or submission, redundancy, etc.) have been completely observed by the authors.
